# Figure-of-Eight Cerclage With High-Resistance Sutures Plus InternalBrace as Treatment for Posterior Dislocation of the Sternoclavicular Joint: A Case Report

**DOI:** 10.7759/cureus.64400

**Published:** 2024-07-12

**Authors:** Jose L Millet-Herrera, Adrián Pérez-Navarrete, Ermilo Echeverría-Ortegón, Ricardo Alejos-Gómez, Nina Mendez-Dominguez

**Affiliations:** 1 Clinical Sciences, Universidad Marista de Mérida, Mérida, MEX; 2 Orthopedic Surgery, Centro Médico de las Américas, Mérida, Yucatán, MEX; 3 Research and Learning, Hospital Regional de Alta Especialidad de la Peninsula de Yucatan, Merida, MEX

**Keywords:** internalbrace, figure-of-eight cerclage, open reduction, posterior sternoclavicular joint dislocation, sternoclavicular joint dislocation

## Abstract

The sternoclavicular joint dislocation is a very infrequent injury that can put the patient's life at risk if it is not diagnosed and treated properly. This can present as an anterior or posterior dislocation, the latter being less common and more dangerous due to its proximity to visceral structures of the thoracic cavity. Herein, we present the case of a 19-year-old male athlete diagnosed with a posterior dislocation of the right sternoclavicular joint due to indirect trauma during a soccer match, who was successfully treated with a figure-of-eight cerclage with high-resistance sutures plus an InternalBrace technique. After recovery, he has been able to get back to sports with a complete range of motion and experiencing no instability after a two-year follow-up. Figure-of-eight cerclage with high-resistance sutures plus an InternalBrace could be a good technique for surgical treatment of this rare injury, especially in young and physically active patients.

## Introduction

The sternoclavicular joint (SCJ) is remarkably stable due to its component ligaments and limited range of motion. The integrity of the SCJ is vital to maintain the stability of the chest wall, as well as for adequate mobility of the shoulder girdle, contributing by rotating the clavicle on its longitudinal axis, as well as by raising the SCJ to 40º [1]. The dislocation of the SCJ is an infrequent injury that represents around 1% of all joint dislocations, and they are classified into anterior and posterior dislocations, the posterior being nine times less frequent [2,3]. Dislocation injuries commonly derive from automobile or even sports accidents [4]. The dislocation mechanism can originate from direct trauma to the anteromedial portion of the clavicle, or indirectly, through a force exerted on the posterolateral portion of the shoulder, forcing the medial portion of the clavicle posteriorly [3].

Posterior dislocations, despite being rare, can have life-threatening complications, ranging from compression or tearing to damage to the subclavian vessels, brachial plexus, vagus and recurrent laryngeal nerve, larynx, esophagus, and lungs; for this reason, an adequate diagnosis and treatment with reduction of the lesion are necessary to avoid the development of complications [2,5]. The surgical management of posterior sternoclavicular dislocations can be controversial given the scarcity of scientific evidence and the consequent limited experience of orthopedic surgeons in the management of this rare pathology [5]; although there are various alternatives for open reduction of the lesion, a gold standard has not yet been defined [1]. Herein, we present the case of a 19-year-old athlete who came to the emergency room with a posterior dislocation of the right SCJ due to indirect trauma, successfully treated by figure-of-eight cerclage with high-resistance sutures together with an InternalBrace. The objective of the article is to describe the diagnosis and surgical management that was carried out for this rare injury.

## Case presentation

**Figure 1:**
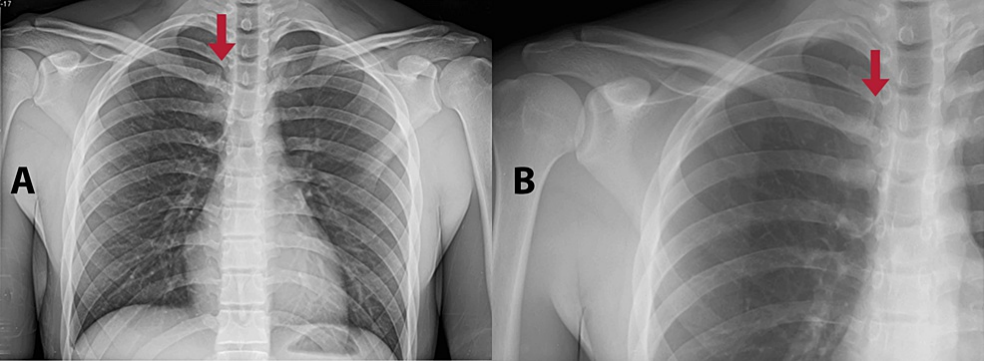
Radiographic images demonstrate deviation in the medial part of the right clavicle. (A) Anteroposterior chest radiography. (B) Right shoulder radiography.

**Figure 2:**
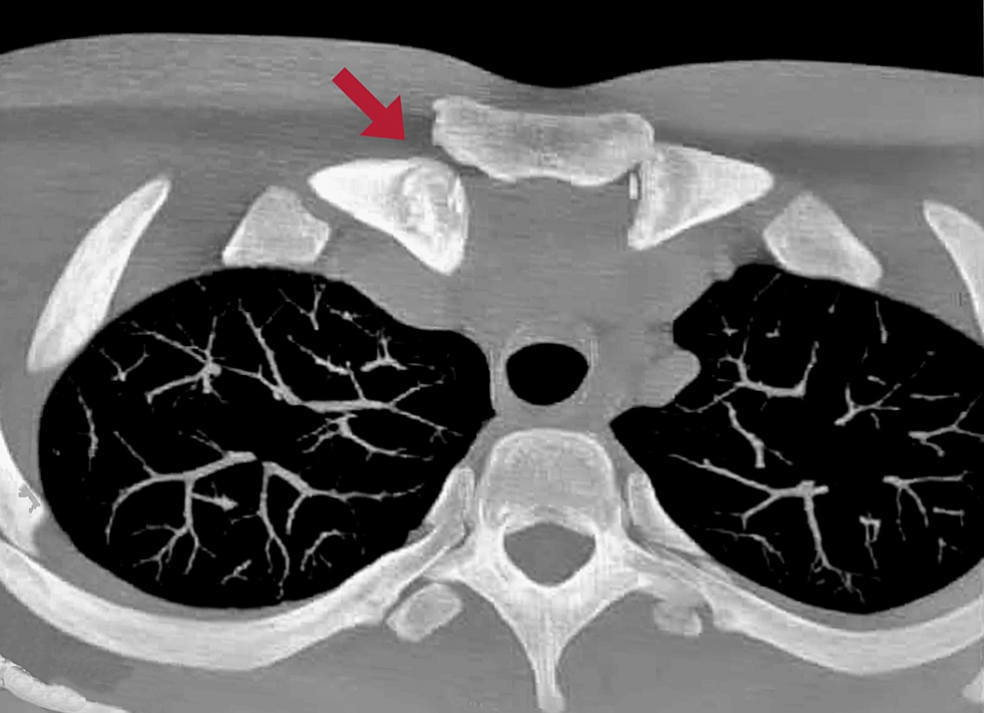
Chest computed tomography showing posterior dislocation of the right sternoclavicular joint.

A 19-year-old male, college student, and high-performance athlete (mainly soccer and gymnasium), presented to the emergency room with pain in the upper right chest, which arose from an accident during a soccer game, in which the patient suffered a fall on the posterolateral part of his right shoulder, and the rival fell on the contralateral shoulder. The patient, who is 1.75 m tall and weighs 62 kg, has no relevant family history. During examination, an x-ray ruled out the presence of a fracture, and he was discharged with analgesics. However, the next day he went to the emergency room of another medical center due to persistent pain. On physical examination, edema in the upper right chest region and tenderness were noted, along with inability to perform movements due to intense pain that worsens with abduction, flexion, and extension of the shoulder. In a new chest x-ray (Figure 1A) and shoulder radiography (Figure 1B), a deviated medial part of the clavicle was observed, and no fractures were seen. A chest computed tomography was conducted (Figure 2), where the presence of a posterior deviation of the SCJ was identified, with which the diagnosis of posterior dislocation of the SCJ was made.

After the interdisciplinary analysis of the case, in the pre-surgical planning, an open reduction with internal fixation was decided using the InternalBrace® (Arthrex, Inc., Naples, FL) technique with two 3.5 mm SwiveLock® (Arthrex, Inc., Naples, FL) anchors and high-resistance FiberTape® sutures (Arthrex, Inc., Naples, FL), accompanied by a figure-of-eight cerclage, also with FiberTape®. An anterior approach was made, with access through a longitudinal incision of approximately 10 cm to visualize the manubrium of the sternum, up to the junction of the distal and middle thirds of the clavicle. Subsequently, a spacer was placed on the posterior face of the medial part of the clavicle, and two 2.5 mm holes were made with the drill, located in an anterior-superior and anterior-inferior position, 1 cm lateral to the medial end of the clavicle. Two holes (also 2.5 mm) were added 1 cm medial to the lateral border of the manubrium of the sternum. Once the four holes were made, the high-resistance suture was introduced through them to achieve a figure-of-eight cerclage, making sure that the SCJ was in a reduced position when tying the sutures. Two more holes were made (3.5 mm each), one placed 2 cm from the medial end of the clavicle in an anteroposterior direction, and another one located 2 cm from the lateral end of the clavicular border of the manubrium sternum, then the anchors were loaded with the FiberTape® sutures and inserted into both holes and tightened. Subsequently, the mobility of the joint was evaluated, and the wound was closed in layers.

**Figure 3:**
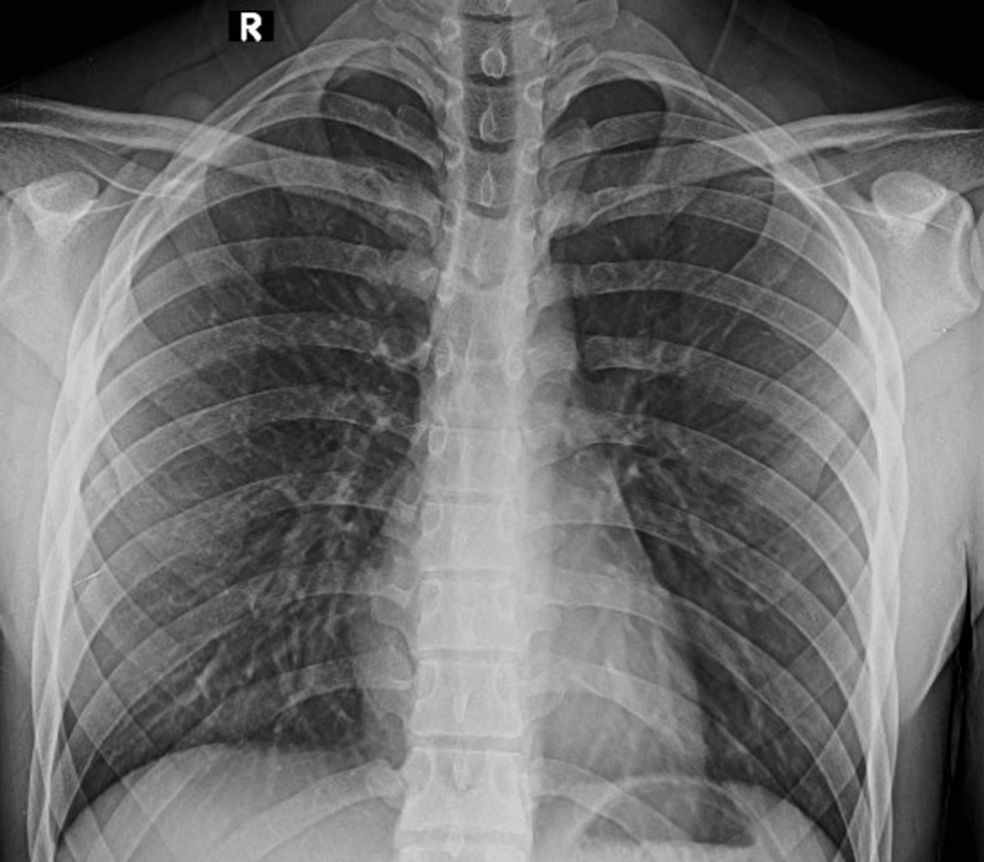
Anteroposterior chest x-ray two years after the surgical procedure showing no abnormalities in the sternoclavicular joint.

The patient had a trans and post-surgical procedure without eventualities, he recovered adequately, beginning rehabilitation two and a half weeks after surgery, with passive mobilization for another two weeks. He returned to his usual sports activities without limitation of function or mobility four months after the event. One year after the surgical procedure, the patient denies pain or symptoms related to any involvement of nerves or blood vessels has a full functional capacity with a DASH score of 0% and maintains the level of physical activity and sports habits as before the accident. After two years, the patient is still without complaints, and he has not experienced any instability during his normal sports activities. A new chest x-ray shows no abnormalities (Figure 3).

## Discussion

We have presented for the first time the combined management of figure-of-eight cerclage with high-resistance sutures together with InternalBrace for the reduction of a posterior sternoclavicular dislocation. Although the lack of a gold standard for the management of these dislocations justifies the communication of this technique, the limitations derived from the experience in a single patient must be taken into account, this can be considered as a proposal for surgical resolution. This type of technique could not be applied if the patient's conditions were different, such as in the presence of polytrauma that complicates the approach or in coexistence with comorbidities.

Regarding diagnosis, it is generally accepted that the imaging study of choice for the diagnosis of sternoclavicular dislocations is computed tomography [3], which allows for determining the exact position of the medial end of the clavicle with respect to the sternum, in addition to offering the possibility of ruling out physical injuries [5]. Due to the potential risk of complications, posterior dislocations must be reduced in all cases; due to the lack of success of closed reductions (successful in only 38% of cases) [6] and the possibility of iatrogenicity during the maneuvers [7], open reduction and surgical fixation performed early may be suitable. Different techniques have been described for open reduction of SCJ dislocations, such as fixation with plates or figure-in-eight cerclage with tendon grafts; however, a gold standard for open reduction of these injuries has not yet been established [1]. Due to the mobility that the SCJ confers to the shoulder girdle, the rigid behavior of the metal plates makes their use inconvenient for the management of these patients, in addition to the possibility that they have to be removed later [1]. The use of tendon grafts does not have this problem since they respect the mobility of the joint; however, the comorbidity associated with the use of tendon grafts, as well as the failures associated with degeneration, rupture, or elongation of the tendon, give this technique certain limitations [1].

In this case, for the management of the posterior sternoclavicular dislocation, a figure-of-eight cerclage was performed with high-resistance sutures together with an InternalBrace, which is a procedure composed of two techniques that have not been reported in the literature being used together as a treatment for sternoclavicular dislocations. Figure-of-eight cerclage with high-resistance sutures is a technique that respects the mobility of the SCJ, which preserves the range of motion of the shoulder girdle while eliminating the aforementioned limitations of tendon grafts; the use of high-resistance sutures in the figure-of-eight cerclage provides sufficient strength and flexibility to replace the function of the damaged ligaments, in addition to providing greater durability than the other techniques described [1]. On the other hand, the InternalBrace consists of a ligament repair technique in which an ultra-high molecular weight polyethylene/polyester braided suture is used, which increases joint strength by acting as a secondary stabilizer, which enables a faster return to sport, since it allows joint mobilization from an early phase of rehabilitation, and also helps to resist injuries and prevent recurrences [8]. The InternalBrace has been used successfully as a treatment for anterior dislocations of the SCJ [8], in addition to having been shown to be effective for the management of different ligament injuries of the knee, shoulder, and elbow [9]. Due to the patient's lifestyle, which is very active due to his sports activities, the use of the InternalBrace was considered adequate as a secondary support to the cerclage, due to the aforementioned benefits.

With respect to the procedure, it is important to highlight that a separator must be placed on the posterior face of the medial end of the clavicle, to protect the vascular and nervous structures found there [7], since their damage with the drill could have fatal consequences. The holes for the figure-of-eight cerclage were made 1 cm medial to the lateral edge of the manubrium of the sternum, and 1 cm lateral from the medial end of the sternum, to avoid cortical rupture [7]. The holes for the InternalBrace were placed at 2 cm so that they would not coincide with those already mentioned. The literature mentions that one of the factors with the greatest influence on the success of open reduction is the time since those patients with better functional results are those operated on early [10], which is consistent with the management of the present case, in which the reduction was performed within a window of 24 hours post-trauma. The main limitation of the implementation of this technique lies in the need to have the necessary tools and materials available.

## Conclusions

In conclusion, posterior SCJ dislocations are very infrequent injuries, and figure-of-eight cerclage with high-resistance sutures together with an InternalBrace may be a suitable alternative as a treatment for single posterior dislocation of the SCJ in patients with no significant pathological history, especially in young and physically active patients, since it makes it possible to compensate for some of the disadvantages of other surgical techniques reported in the literature, and offers a quick and safe return to sports activity; nevertheless, further research is required to confirm the efficacy of this procedure as a therapeutic option for posterior dislocation of the SCJ.
